# Medicines postpartum in Sweden and coverage in Janusmed Breastfeeding

**DOI:** 10.1007/s00228-023-03528-x

**Published:** 2023-07-15

**Authors:** A. B. Asplund, P. Dreher Sköld, L. Karlsson Lind, C. E. Cesta, M. L. Dahl, E. Wikström Jonsson, M. L. Andersson

**Affiliations:** 1 Clinical Pharmacology, Department of Laboratory Medicine, Karolinska Institutet, Karolinska University Hospital, Stockholm, Sweden; 2 Department of Knowledge Development, Health and Medical Care Administration, Region Stockholm, Sweden; 3 Department of Medicine Solna, Centre for Pharmacoepidemiology, Karolinska Institutet, Stockholm, Sweden

**Keywords:** Medicines, Postpartum, Janusmed breastfeeding, Breast feeding milk, Human cohort studies decision support systems, Clinical database management systems

## Abstract

**Purpose:**

The purpose of this article is (1) to investigate which medicines are prescribed and dispensed to women the first 6 months postpartum, (2) to identify medicines dispensed postpartum but not recommended during breastfeeding, and (3) to find medicines commonly dispensed postpartum, but not currently included in Janusmed Breastfeeding.

**Methods:**

In this register-based cohort study covering births between January 2017 and August 2019, the Swedish Medical Birth Register (MBR), the Prescribed Drug Register, and Janusmed Breastfeeding were linked to identify medicines dispensed to women during the first 6 months postpartum, and how they are covered and classified in Janusmed Breastfeeding.

**Results:**

During the first 6 months postpartum, 66% of women purchased at least one prescription medicine from the pharmacy. The most common medicines were contraceptive agents, analgesics, antibiotics, and glucocorticoids. A third of the 30 most commonly dispensed medicines have no information available about the safety of use in breastfeeding. The most dispensed medicines, where the database advises against use in breastfeeding, included several antitussive agents, a local anaesthetic, and several gestagens. The most commonly dispensed medicines not covered by the Janusmed Breastfeeding were medicines for dry eyes, for assisted reproduction, and HIV.

**Conclusion:**

Prescribed medicines compatible with breastfeeding are more common during the first 6 months postpartum than medicines not compatible with breastfeeding, but medicines which lack evidence for safety in breastfeeding are still commonly used.

**Supplementary Information:**

The online version contains supplementary material available at 10.1007/s00228-023-03528-x.

## Introduction

In Sweden, almost all women start breastfeeding after they have given birth. The prevalence of breastfeeding gradually decreases to 83% at 2 months, 73% at 4 months, 60% at 6 months, and 30% at 1 year postpartum [1]. The decrease during the first 6 months, when WHO recommends full breastfeeding, is likely multifactorial and may in part be due to breastfeeding difficulties, but worries of medicine exposure in the child through breast milk might also contribute. Easily accessible information on whether medicines pass into breast milk and whether they can have a pharmacological effect on the breastfed child is therefore crucial. Janusmed Breastfeeding (Janusmed Amning in Swedish), a computerised decision support system (CDSS) providing evidence-based information concerning the risks associated with exposure to medicines via breastmilk, was developed to meet this need. However, this CDSS does not cover all medicines available in Sweden. Only medicines presumed to be used by potentially breastfeeding women have been added.

Historically, when prescribers or breastfeeding women have needed medicines information, the most common source has been FASS (the Swedish Physicians’ Desk Reference) which is based on the official Summaries of Product Characteristics (SmPC) [2]. However, as the SmPCs are written by the manufacturers who are not required to conduct human lactation studies, or literature searches to find information published by others, these texts often lack information on passage of medicines into human milk and on the effects on breastfed infants even when such data exist. Instead, animal data are often used but this may not accurately reflect medicine transfer in humans as the composition of breast milk differs greatly between species [3].

The WHO recommendation to breastfeed for at least 6 months is based on benefits for both the infant and the mother. For the infant, a decrease in the risk of respiratory tract infections, gastroenteritis, inflammatory bowel disease, sudden infant death syndrome, atopic dermatitis, and obesity has been proposed. For the mother, the benefits include decreased postpartum blood loss, more rapid uterus involution, and lower fertility in the short-term as well as decreased risk for osteoporosis and for breast and ovarian cancer in the long-term [3].

On the other hand, there are case reports of serious adverse effects in breastfed infants attributed to pharmacological treatment of the mother [4, 5], which is why a careful risk-benefit evaluation must be performed for each medicine.

In 1987, the Drug Information Centre (Karolic), at the Department of Clinical Pharmacology, Karolinska University Hospital, Huddinge was contacted by the Department of Paediatrics with a request of a list with evaluated information on medicines commonly used in breastfeeding women. The list was made, regularly updated, and 10 years later, it was converted to a database which today contains almost 900 documents with breastfeeding recommendations. The database is freely available and published as the CDSS Janusmed Breastfeeding on https://janusmed.se/amning. It is also implemented in most electronic health record systems in Sweden and automatically signals warnings if women between the ages 13 and 55 are prescribed medicines not recommended during breastfeeding (see Table 1). The database is also implemented in the decision support system available for Swedish pharmacies (EES) [6].

**Table 1:**
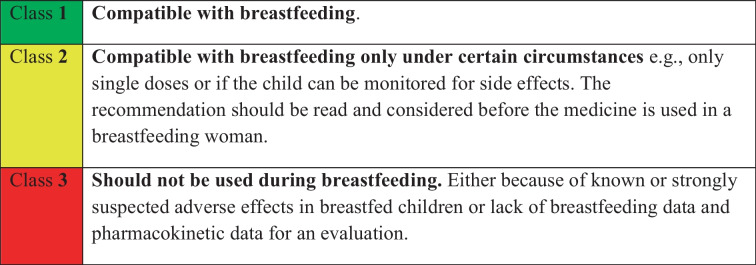
Classification of risk for a breastfed child

Freely available information sources in the breastfeeding field such as the Drugs and Lactation Database LactMed [7] exist but the texts in these sources are written in English and too extensive for use in the prescribing situation. The prescriber needs short, easily comprehendible information, available in the health record system [8] and preferably clearly stating a yes or no for use during breastfeeding.

The purpose of this article is (1) to investigate which medicines are prescribed and dispensed to women the first 6 months postpartum, (2) to identify medicines dispensed postpartum but not recommended during breastfeeding, and (3) to find medicines commonly dispensed postpartum, but not currently included in Janusmed Breastfeeding.

## Janusmed Breastfeeding

Janusmed Breastfeeding is produced in collaboration between the Department of Knowledge Development, Health and Medical Care Administration, and the Department of Clinical Pharmacology, Karolinska University Hospital, Region Stockholm, Sweden. The database is made available by Region Stockholm and financed nationally. The content in Janusmed Breastfeeding is written by pharmacists and physicians in clinical pharmacology and approved by a specialist in clinical pharmacology. The database is updated four times per year.

The working process includes eight steps as described in (Table 2).

**Table 2:** The working process for Janusmed Breastfeeding

**Step**		
**1**		A list of substances to be considered for an update is made based on:
	*a*	A standardised updated literature search in PubMed (see Appendix 1)
	*b*	Newly approved medicines
	*c*	Requests from users of the database
	*d*	Review of all documents in a pharmacological group
	*e*	Review of current linkage to medicinal products
**2**		A meeting with all involved in the maintenance of the database where substances to be added or updated are chosen
**3**		A full literature search is made for the chosen substances (see Appendix 1)
**4**		A new text (see Fig. 1) is written by a pharmacist or physician and entered in the database. The text incudes passage into breast milk, relative infant dose, and an assessment of the infant risk based on outcome for breastfed infants and pharmacological reasoning including the metabolising capacity of infants (see Medicines and breastfeeding -aspects to consider). The database is linked to The National Repository for Medical Products [9] covering all medicines in Sweden, and The National Substance Register for Medicinal Products [10], containing information about active substances
**5**		A meeting with all the pharmacists or physicians and a specialist in clinical pharmacology discusses the new texts, their classification, and the recommendations (see Fig. 1)
**6**		The texts are counter signed by a specialist in clinical pharmacology
**7**		The final texts are proofread and then published by pharmacists at the Health and Medical Care Administration
**8**		New medicinal products/drug forms entering on the Swedish market are evaluated and linked to published documents on a regularly basis

As the information on passage into breast milk and effects on a breastfed infant is absent or very limited for most medicines, it has been necessary to implement three classes (Table 1) for the evaluation of the risk for a breastfed infant.

When little or no information is available, an evaluation is made based on the pharmacokinetic and pharmacodynamic properties of the medicine. The degree of evidence is shown as a grade of documentation (Table 3).

**Table 3:** Level of documentation for use in breastfeeding

**0 = **	Clinical documentation is lacking
**1 = **	A few case reports, unpublished data, or substandard studies
**2 = **	Studies on transport into breast milk and/or plasma concentrations in breastfed children or effects in breastfed children

More than 100,000 searches are made in Janusmed Breastfeeding annually. In a survey of users of the web version of the database in 2016, about a third of the users were patients or their relatives and the other two thirds were professionals (physicians, midwives, nurses, and pharmacists) [an unpublished survey made to see who is using the database and find out what they think of it]. The medicines most commonly searched for are analgesics (ibuprofen, diclofenac, paracetamol, naproxen), antidepressants (sertraline), antihistamines (loratadine, desloratadine), nasal decongestants (xylometazoline), medicines for treatment of rhinitis (mometasone), and medicines to prevent nausea and vomiting in pregnancy or as hypnotics (promethazine).

The database is limited to advice for the use of medicines in women breastfeeding healthy, full-term infants. For breastfeeding of sick or preterm infants, the Swedish users are advised to contact their regional medicines information centre (https://svelic.se/om-lic/) for an individual evaluation of the available information.

### Medicines and breastfeeding — aspects to consider

#### Passage into breast milk

To affect a breastfed child, the mother’s medication must pass into breast milk. During the colostral phase, the spaces between the epithelial cells in the mammary gland allow large molecules, such as immunoglobulins, to pass, but about a week postpartum, these spaces close allowing only very small molecules of less than 200 Daltons through, while all larger molecules must pass from plasma to breast milk through passive diffusion or active transport over the membrane. However, only a few medicines have been shown to be actively transported into breast milk, e.g. aciclovir and methotrexate [3].

As breast milk has a slightly lower pH than plasma, weak bases usually accumulate in higher concentration in breast milk than in plasma and weak acids are normally found in lower concentrations in milk than in plasma. Breast milk contains more fat than plasma, especially the last milk expressed in a feeding. The fat content in milk varies over the day. Medicines which are highly protein bound pass into breast milk in a lower degree. As the passage into milk by passive diffusion is driven by the concentration gradient, the direction of the flow changes when the concentration in the mother’s plasma falls below the concentration in breast milk [3].

#### Elimination capacity in infants

A new-born infant has higher intra-gastric pH, slower gastric emptying, different gastrointestinal microflora, and higher proportion of body water than older children and adults. Furthermore, their drug-metabolising enzyme system and renal function are immature [11]. Therefore, the decreased elimination capacity increases the risk of accumulation of medicines in the infant. This risk is even higher for premature infants.

#### Relative infant dose

For medicines with a well-established infant dose, this dose can be used as a reference for assessment whether the dose transferred through breastmilk is close enough to pose a risk for a breastfed infant. However, for most medicines used by breastfeeding women, no established dose for infants exists. In these cases, the dose the breastfed child is exposed to is instead compared to the dose of the mother. As the child is so much smaller than its mother, the comparison is done as dose per kg body weight and is known as “Relative Infant Dose”. It has been proposed that a relative infant dose of less than 10% indicates that the medicine is safe for use in breastfeeding women [3, 12]. However, the limit of 10% has been chosen arbitrarily. Naturally, 10% of a therapeutic dose of a vitamin is not directly comparable to 10% of a cytostatic agent. Instead, an evaluation must be made for each medicine based on its pharmacological profile. For medicines in the same therapeutic class, the relative infant dose can be one of the factors to consider when choosing the best therapeutic alternative for a breastfeeding woman.

There are several ways of calculating a relative infant dose and for Janusmed Breastfeeding, it is always calculated by the Janusmed team, using the formula below, where the infant dose per kg is divided by the mother’s dose per kg (${D}_{Mother\, (mg/day)}/{M}_{Mother\, (kg)}$) giving a relative infant dose in percent (${D}_{Infant}$). As the dose to the child is not known, this in turn is calculated from the concentration in the milk at steady state (${C}_{SS(mg/mL)}$ and the volume of milk consumed by the infant (${V}_{Milk (mL/kg/day)}$).

$${D}_{Infant}=\frac{{C}_{SS(mg/mL)}\cdot {V}_{Milk \, (mL/kg/day)}\cdot {M}_{Mother\, (kg)}\cdot 100}{{D}_{Mother (mg/kg/day)}}\mathrm{\%}$$

If no data is provided concerning milk intake or the mother’s weight, a milk intake of 150 mg/kg/day and a weight of the mother of 60 kg are assumed. The relatively low weight of the mother is chosen to find the worst-case scenario.

In cases where only single dose data exist, an extrapolation from single dose data to average concentration at steady state conditions ($${C}_{SS})$$ is made. This is to avoid a false low relative infant dose which would otherwise be the case for medicines which are used therapeutically in repeated doses, and which have not been fully eliminated when the next dose is taken. The would-be concentration at steady state can either be calculated from the area under the concentration-time curve ($${AUC}_{ng/mLh}$$) for the dosing interval ($${\tau }_{h}$$)

$${C}_{SS}=\frac{{AUC}_{ng/mLh}}{ {\tau }_{h}}$$

or from the average concentration ($${C}_{av})$$ using a cumulation factor ($$R$$) ($${C}_{SS}={C}_{av}\cdot R$$) which in turn can be calculated from the dosing interval ($$\tau$$) and the half-life ($${t}_{{}^{1}/{}_{2}}$$).

$$R=\frac{1}{1-{e}^{-\tau ln2/{t}_{{}^{1}/{}_{2}}}}$$

A worst-case scenario can be calculated from a maximum concentration (${C}_{max}$) using the same formula (${C}_{SS}={C}_{max}\cdot R$). Either way the (${C}_{SS}$) can then be used to calculate the relative infant dose as above.

## Register-based cohort study

### Method

In this register-based cohort study, we used the Medical Birth Register (MBR) [13] to identify all women in Sweden giving birth to at least one liveborn child between January 2017 and August 2019. The MBR contains data on all pregnancies resulting in a live or stillborn child after 22 weeks gestation. August 2019 was chosen as the end of the study period since we expected the findings to be influenced by the COVID-19 pandemic starting in March 2020 in Sweden [14]. To investigate the use of medicines within the first 6 months after giving birth, we linked the data from MBR to the Prescribed Drug Register (PDR) [15], which covers all prescriptions dispensed at a pharmacy in Sweden. Janusmed Breastfeeding data was downloaded from an application programming interface (API) and linked to patient data.

Characteristics of the pregnant women who delivered liveborn infants are presented as absolute numbers, percentages, and medians as appropriate. The postpartum period of women delivering stillborn infants were not included. The number of medicines dispensed per postpartum period (defined as 6 months after pregnancy) was calculated and the proportion of postpartum periods where the women did not receive any prescription medicine was reported.

The medicines dispensed were translated into active substances and the number of exposures for each substance per postpartum period was summarised in a frequency table including all substances with 100 or more exposures. For example, if a patient gives birth two times within the study period, her medication for 6 months after first delivery and 6 months after the second delivery will be included separately in the analysis. A separate analysis was performed for medicines use during the first week postpartum. We also analysed the most common medicines classified as class 3 (medicines to avoid in lactation), and the most common substances used that were not covered by Janusmed Breastfeeding at the time of analysis (September 2022). For the last two analyses, we included substances and their route of administration (classified according to Janusmed Interactions [16, 17]). All analyses were performed using R 4.0.2 [18].

### Results

Between 1 January 2017 and 31 August 2019, 303,113 pregnancies in Sweden resulted in at least one liveborn child (see Table 4). During the first 6 months postpartum, 188,313 (66%) women purchased at least one prescribed medicine (Fig. 2). In total, prescriptions of 667 different substances were dispensed during the study period. The most dispensed substances belonged to the contraceptive agents, analgesics, antibiotics, and glucocorticoids drug classes (Table 5). Low molecular heparins (dalteparin, tinzaparin) and hypertensive agents, specifically labetalol, were commonly dispensed during the first week postpartum (Table 6). Of the 30 most dispensed substances, none were class 3; however, a third of these substances lack information on passage into breast milk as well as effects on breastfed infants, e.g. antibiotics and glucocorticoids. The most commonly dispensed medicines within class 3 in Janusmed Breastfeeding include a local anaesthetic, several antitussive agents, and several gestagens (Table 7). Of the 863 documents available in Janusmed Breastfeeding, 53% (*n* = 457) lack information on passage into breast milk and effects on breastfed infants. The substances most often used but not included in Janusmed Breastfeeding were medicines for dry eyes, assisted reproduction, and HIV (Table 8).

**Table 4:** Description of the study cohort

**The cohort**	
Number of women	285 308
Number of pregnancies with liveborn child	303 113
The age of the mother year (median, IQR)	30 (27–34)
Number of medicines dispensed in the 6 months postpartum (mean)	1.41
Number of medicines dispensed in the 6 months postpartum (median, IQR)	1 (0–2)

**Fig. 1:**
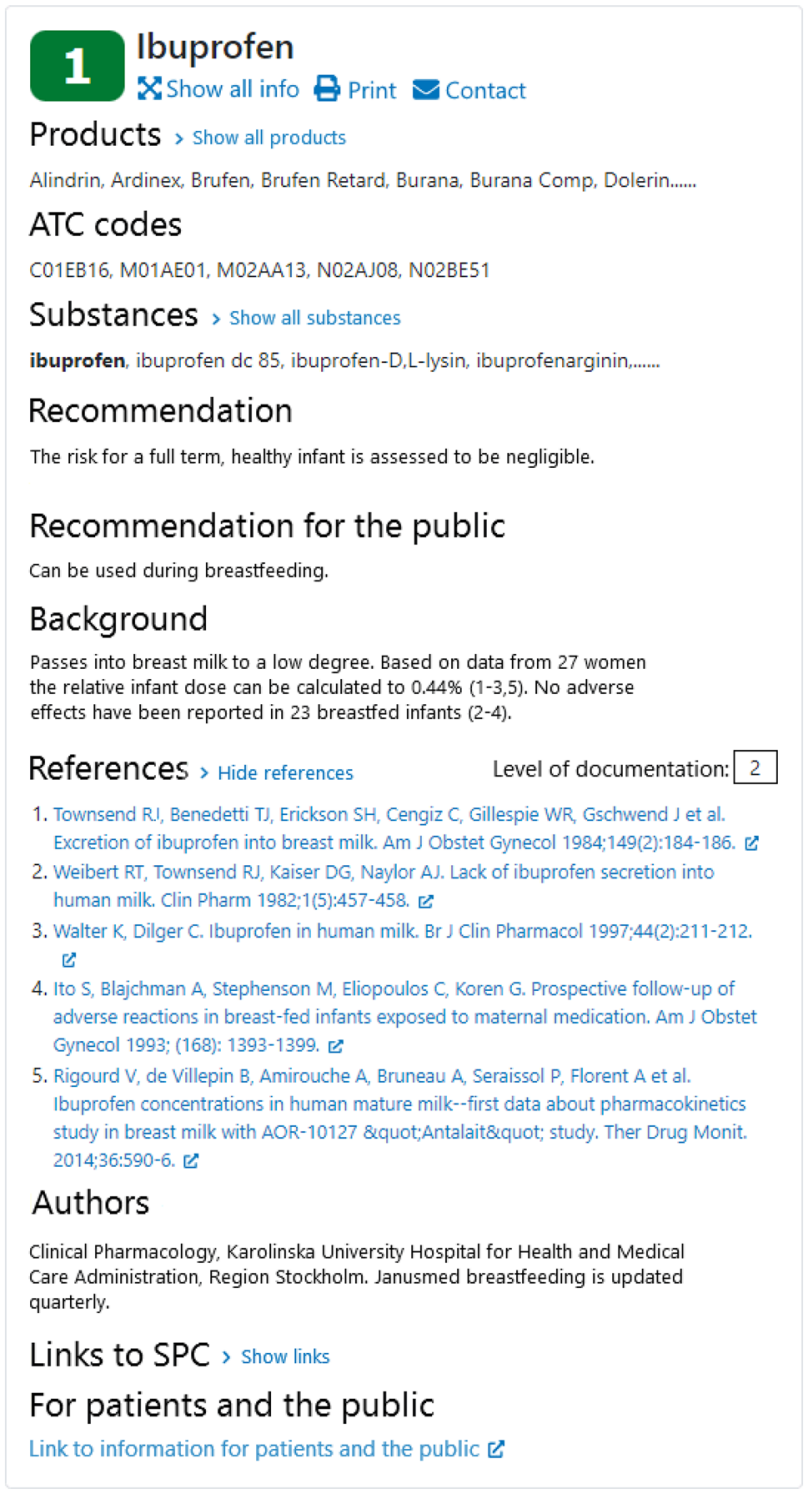
An information card from Janusmed Breastfeeding, translated into English

**Fig. 2:**
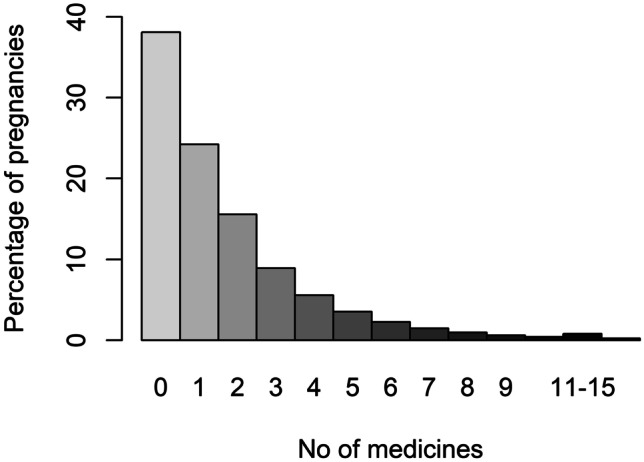
Number of prescription medicines dispensed per pregnancy during the first 6 months postpartum

**Table 5:**
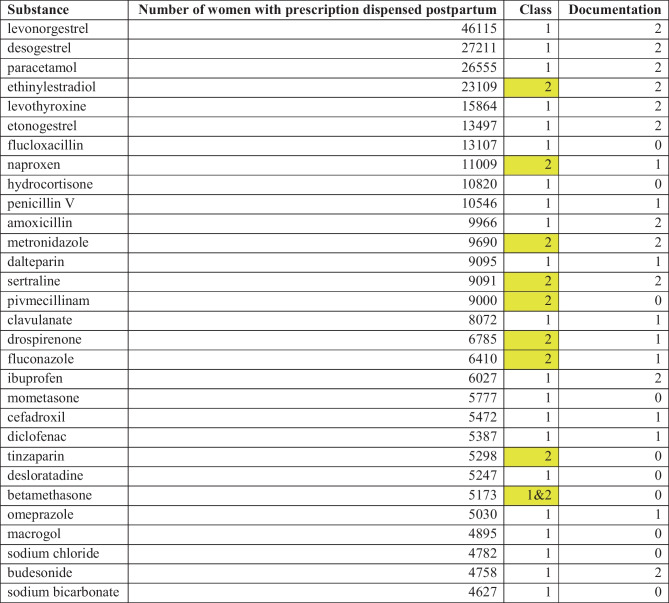
Top 30 most commonly dispensed medicines to women during the first 6 months postpartum. The medicines with class 2 are marked with yellow in the table in accordance with the colour-coding in Janusmed Breastfeeding (Table 1)

**Table 6:**
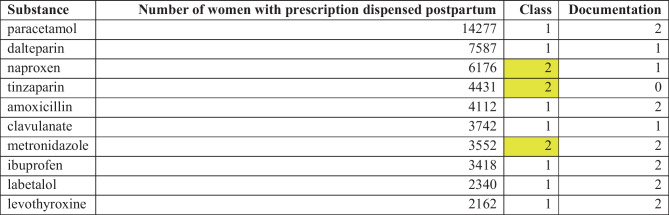
Top 10 most commonly dispensed medicines to women the first week postpartum. The medicines with class 2 are marked with yellow in the table in accordance with the colour-coding in Janusmed Breastfeeding (Table 1)

**Table 7:** Top 10 most commonly dispensed class 3 substances to women during the first 6 months postpartum

**Substances with class 3**	**Number of women with prescription dispensed postpartum**
Cinchocaine	2185
Ethylmorphine	1819
Cocillana extract	1761
Senega extract	1761
Norgestimate	1711
Medroxyprogesterone — high dose	1560
Bromhexine	1516
Propiomazine	1185
Norelgestromine	1145
Chlorzoxazone	1126

**Table 8:** Substances dispensed to women postpartum but missing in the database Janusmed Breastfeeding

**Substance**	**Administration form**	**Number of women with prescription dispensed postpartum**
Carboxypolymethylene	Topical	321
Hypromellose	Topical	128
Abacavir	Oral	106
Lamivudine	Oral	106
Dolutegravir	Oral	95
Emtricitabine	Oral	95
Povidone K25	Topical, eye drops	51
Darunavir	Oral	45
Ganirelix	Parenteral	39
Efavirenz	Oral	38
Follitropin alfa	Parenteral	35
Aprepitant	Oral	23
Atazanavir	Oral	21
Nafarelin	Topical	20
Phosphoric acid	Oral	17
Filgrastim	Parenteral	17
Dextran 70	Topical, eye drops	16
Neomycin	Topical	16
Cobicistat	Oral	15
Menotropin	Parenteral	14
Quinagolide	Oral	12
Sodium selenite	Oral	12
Trihexyphenidyl	Oral	11
Betahistine	Oral	10

For more data on medicines dispensed to women in the first 6 months postpartum, see Appendix 2.

## Discussion

Information on which medicines are used in breastfeeding women and the effect of these medicines on breastfed infants are largely missing [3]. Of the documents included in Janusmed Breastfeeding, 53% lack information both on passage into breast milk and effects in breastfed infants. Half of the medicines with class 3 have this classification due to insufficient pharmacokinetic information.

In the present study, we summarise the prescription substances dispensed to women within 6 months postpartum in Sweden. A Swedish study published in 2011 [19] similarly reported medicines dispensed during the postpartum period but data was presented at a pharmacological group level and not as single substances. Hence, the study could not be used as a basis for updating Janusmed Breastfeeding. Therefore, we conducted this study, in part, to further improve the coverage of the CDSS Janusmed Breastfeeding, by including substances used in the postpartum period in the Swedish population but not previously covered by the database.

We chose to include medicines data for 6 months postpartum as this is the period when most women breastfeed according to national statistics [1]. The Swedish registers do not contain individual level data on breastfeeding which is a limitation because we do not know which of the women in our study population actually breastfed their children. Hence, we also include medicines used by non-breastfeeding mothers in our analysis. An advantage of this approach is that it provided us with large data (more than 300,000 pregnancies) including the entire population of Sweden. One must also bear in mind that apart from the prescription medicines, women may purchase over-the-counter medicines or be treated with medicines in the hospital setting, neither of which will be registered in the PDR. For some common medicines, the over-the-counter sales overshadow the prescription sales. An example is paracetamol which has an annual sale of about 18 million packages [20] in the Swedish population of almost 10.5 million [21] giving an average of 1.7 packages per person and year. No statistical data is available on the byers of over-the-counter medicines.

The most used medicines in the first 6 months postpartum were contraceptive agents, analgesics, antibiotics, and glucocorticoids, which is in accordance with previously published studies [19, 22, 23]. None of the medicines in the top 30 list were class 3 substances, but whether this is attributable to the availability of Janusmed Breastfeeding in the electronic health record systems or not is unknown. However, the warning with a red sign every time a medicine unsuitable for breastfeeding is prescribed should be a reminder to the prescriber to consider the pros and cons of medicines use in that patient. Interestingly, a third of the medicines in top 30 list lack published information about passage into breast milk as well as effects on the breastfed infant.

For some of the medicines with breastfeeding class 3, there are alternatives with more documentation or lower passage into breast milk. Some examples where class 3 substances have been used although there are alternatives with class 1 are the local anaesthetic cinchocaine available in the medicine for haemorrhoids (alternative lidocaine), the antihypertensive losartan (alternative candesartan), the proton pump inhibitor pantoprazole (all other proton pump inhibitors), and the laxative laurylsufloacetate (alternatives bisacodyl, docusate, and sorbitol). Some of the treatments, such as the antitussive agents, lack both evidence for their clinical efficacy [24] and safety data for breastfeeding but are still commonly used. For other medicines like lithium, good alternatives are not available. However, a recent study has shown that lithium can be safely used in highly motivated well informed breastfeeding women if the children are carefully monitored by plasma concentration measurements as well as close clinical follow-up [25]. As this routine may not be possible in all parts of Sweden, and not all women with bipolar disease are able to follow these instructions, the general recommendation to avoid lithium in breastfeeding remains, with a comment on how to monitor if breastfeeding is chosen.

In top of the list of medicines missing in Janusmed Breastfeeding are three therapeutic groups: (1) medicines for dry eyes, (2) medicines for assisted reproduction, and (3) HIV-medicines. The two first groups will be added to the database to increase its usability, but the HIV-medicines will not be added since breastfeeding is not permitted for HIV-positive women in Sweden according to the Swedish Communicable Diseases Act [26].

## Supplementary Information

Below is the link to the electronic supplementary material.

Supplementary file1 (PDF 29 KB)

Supplementary file2 (PDF 100 KB)

## Data Availability

The datasets can be accessed from the authors if the requester has a valid ethics approval.
