# Supplementary material for: Medicines postpartum in Sweden and coverage in Janusmed Breastfeeding
Source: Eur J Clin Pharmacol. 2023 Jul 15;79(9):1261–9. doi: 10.1007/s00228-023-03528-x (PMC10427528; doi:10.1007/s00228-023-03528-x)
Supplement: Supplementary file 1 — Supplementary file1 (PDF 29 KB) [file 228_2023_3528_MOESM1_ESM.pdf]

Appendix 1

Standardised literature search.

Every third month a screening search in PubMed is done using our standardised literature search:

"milk, human"[MeSH Terms] OR "breastfeeding"[MeSH Terms] OR "breastfeeding"[All Fields] OR "breastfeeding"[All Fields] OR "lactation"[MeSH Terms] OR "lactation"[All Fields] OR "colostrum"[MeSH Terms] OR "colostrum"[All Fields] OR "Breast Milk"[All Fields] OR breastmilk[All Fields]

Full literature search made for the chosen substances

When we are writing texts for a chosen substance, we make a full literature search for that specific substance in the following sources:

Databases

- PubMed
- Embase
- Drugline
- Relis
- LactMed

Books

- Briggs GB, Freeman RK. Drugs in pregnancy and lactation. 10th ed. Philadelphia: Lippincott Williams & Wilkins; 2015.
- Bennett, Drugs and human lactation.2nd ed. Amsterdam; Elsevier 1996
- Schaefer, Drugs during pregnancy and lactation 3rd ed. 2015, Dollery; Therapeutic drugs 2nd ed. 1999
- Avery´s Drug treatment. 4th ed. Auckland: Speight TM, Holford NHG ,Adis; 1997, DRUGDEX® System (electronic version). Truven Health Analytics, Greenwood Village, Colorado, USA
