# Supplementary material for: Medicines postpartum in Sweden and coverage in Janusmed Breastfeeding
Source: Eur J Clin Pharmacol. 2023 Jul 15;79(9):1261–9. doi: 10.1007/s00228-023-03528-x (PMC10427528; doi:10.1007/s00228-023-03528-x)
Supplement: Supplementary file 2 — Supplementary file2 (PDF 100 KB) [file 228_2023_3528_MOESM2_ESM.pdf]

Appendix 2

Table 5. Substances dispensed to at least 100 women during the first 6 months postpartum

Substance	Number of women with prescription dispensed postpartum
levonorgestrel	46115
desogestrel	27211
paracetamol	26555
ethinylestradiol	23109
levothyroxine	15864
etonogestrel	13497
flucloxacillin	13107
naproxen	11009
hydrocortisone	10820
penicillin V	10546
amoxicillin	9966
metronidazole	9690
dalteparin	9095
sertraline	9091
pivmecillinam	9000
clavulanate	8072
drospirenone	6785
fluconazole	6410
ibuprofen	6027
mometasone	5777
cefadroxil	5472
diclofenac	5387
tinzaparin	5298
desloratadine	5247
betamethasone	5173
omeprazole	5030
macrogol	4895
sodium chloride	4782
budesonide	4758
sodium bicarbonate	4627
cholecalciferol	4475
prednisolone	4439
promethazine	4349
codeine	4226
lidocaine	4218
labetalol	3993

oxytocin	3949
clindamycin	3921
cyanocobalamin	3792
sodium feredetate	3631
oxytetracycline	3363
polymyxin B	3264
metoprolol	3262
miconazole	2863
enalapril	2780
escitalopram	2596
sterculia	2566
nitrofurantoin	2557
ephedrine	2463
folic acid	2399
citalopram	2364
oxycodone	2353
urea	2304
salbutamol	2290
calcium gluconate	2250
norethisterone	2240
formoterol	2214
cinchocaine	2186
terbutaline	2181
fusidate	2077
medroxyprogesterone	2072
valaciclovir	2037
insulin human isophan	1932
hydroxyzine	1900
ethylmorphine	1819
sodium fluoride	1815
trimethoprim	1804
estradiol	1796
cocillana extract	1761
senega extract	1761
nifedipine	1714
norgestimate	1711
oxazepam	1577
zopiclone	1551
esomeprazole	1547
bromhexine	1516
sumatriptan	1472
tranexamic acid	1388
fluticasone	1340

venlafaxine	1291
lactulose	1290
fluoxetine	1259
ciprofloxacin	1236
propiomazine	1185
estriol	1163
lamotrigine	1158
morphine	1156
nystatin	1150
norelgestromin	1145
propylene glycol	1136
caffeine	1131
chlorzoxazone	1126
econazole	1118
papaverine	1064
loratadine	1056
sulfamethoxazole	1056
phenylpropanolamine	1013
glycerine	980
chloramphenicol	960
doxycycline	953
bromocriptine	951
dequalinium	932
noscapine	930
zolpidem	894
mesalazine	887
cromoglicate	863
methylscopolamine	835
mebendazole	829
methylphenidate	820
erythromycin	806
clobetasol	804
mirtazapine	804
clemastine	798
metformin	770
lynestrenol	752
nomegestrol	729
triamcinolon	720
azelaic acid	701
dienogest	659
cetirizine	640
clobetasone	628
melatonin	624

sodium picosulfate	610
potassium citrate	606
aciclovir	603
propranolol	589
tramadol	577
aluminium hydroxide-magnesium carbonate	575
azelastine	566
ketoconazole	555
lisdexamfetamine	534
epinephrine	515
alimemazine	513
levocabastine	506
azathioprine	502
acetylsalicylic acid, low-dose	498
dexamethasone	475
amitriptyline	473
quetiapine	463
montelukast	460
duloxetine	459
amlodipine	457
ketoprofen	454
olanzapine	436
diethylamine salicylate	420
buprenorphine	392
metoclopramide	384
calcipotriol	381
diazepam	370
pyridoxine	370
bupropion	366
glyceryl trinitrate	360
naloxone	351
tacrolimus	344
etoricoxib	334
salmeterol	331
adapalene	329
warfarin sodium	326
carboxypolymethylene	321
lithium	317
sodium sulfate	316
benzydamine	314
acetylcysteine	313
furosemide	310

bisacodyl	309
levetiracetam	308
benzoyl peroxide	304
clotrimazole	303
hydroxychloroquine	302
lymecycline	301
olopatadine	287
paroxetine	284
clioquinol	283
terbinafine	281
gabapentin	280
sulfasalazine	267
loperamide	255
zolmitriptan	251
simeticone	243
felodipine	230
beclometasone	228
retapamulin	227
lactic acid	226
sodium citrate	225
alginic acid	219
pregabalin	214
aripiprazole	208
adalimumab	207
ondansetron	203
zinc oxide	200
methotrexate	199
liothyronine	191
clarithromycin	190
enoxaparin	186
cabergoline	181
isoniazid	180
losartan	180
hydrochlorothiazide	175
chlorhexidine	174
fluocortolone	174
sorbitol	173
ascorbic acid	167
hydroxocobalamin	167
carbamazepine	162
tenofovir disoproxil	160
candesartan	157
pantoprazole	151

celecoxib	150
atorvastatin	148
docusate sodium	146
etanercept	143
certolizumab	137
bisoprolol	136
hydralazin	135
prilocaine	135
ranitidine	135
propylthiouracil	133
ispaghula	130
hypromellose	128
pimecrolimus	126
levofloxacin	124
acetylsalicylic acid	122
fexofenadine	121
emedastine	118
ebastine	116
methylergometrine	115
fluocinolone	114
thiamazole	114
amorolfine	113
progesterone	113
lamivudine	112
disulfiram	111
rizatriptan	109
orlistat	108
abacavir	106
azithromycin	103
ivermectin	103
thiamine	101
ketotifen	100

Table 6. Substances dispensed to at least 100 women during the first week postpartum

Substance	Number of women with prescription dispensed postpartum
paracetamol	14277
dalteparin	7587
naproxen	6176
tinzaparin	4431
amoxicillin	4112
clavulanate	3742
metronidazole	3552
ibuprofen	3418
labetalol	2340
levothyroxine	2162
cefadroxil	2006
diclofenac	1919
metoprolol	1851
enalapril	1249
pivmecillinam	1135
macrogol	1091
sodium chloride	980
sterculia	977
sodium bicarbonate	965
codeine	937
lidocaine	913
nifedipine	882
sodium	862
prednisolone	811
oxycodone	708
flucloxacillin	681
cinchocaine	628
hydrocortisone	537
sertraline	506
penicillin V	449
clindamycin	440
lactulose	429
promethazine	385
bromocriptine	315
omeprazole	308
cyanocobalamin	290
trimethoprim	277
ephedrine	248
oxytocin	236

nitrofurantoin	214
buprenorphine	210
insulin human isophan	209
bisacodyl	206
budesonide	200
tranexamic acid	188
valaciclovir	188
sulfamethoxazole	183
bromhexine	180
betamethasone	179
clemastine	177
morphine	174
mometasone	169
desloratadine	163
zopiclone	162
cholecalciferol	154
esomeprazole	149
folic acid	141
citalopram	139
zolpidem	138
caffeine	137
terbutaline	133
lamotrigine	126
escitalopram	123
fluconazole	123
naloxone	115
oxazepam	106
urea	104
calcium gluconate	101
ciprofloxacin	100

Table 7. Substances with class 3 (should not be used while breastfeeding) dispensed to at least 100 women six months postpartum.

Substance	Number women with prescription dispensed postpartum
cinchocaine	2185
ethylmorphine	1819
cocillana	1761
senega	1761
norgestimate	1711
medroxiprogesterone – high dose	1560
bromhexine	1516
propiomazine	1185
norelgestromine	1145
chlorzoxazone	1126
ciprofloxacin - systemic	1016
methylscopolamine	835
nomegestrol	729
dienogest	659
bupropion	366
lithium	317
benzydamine	314
acetylcysteine	313
furosemide	310
methotrexate	199
cabergoline	181
losartan	180
pantoprazole	151
atorvastatin	148
terbinafine - systemic	142
bisoprolol	136
progesterone	113
orlistat	108
azithromycin	103
